# Multiple sclerosis greatly impacts family members/partners: Evidence using the Family Reported Outcome Measure (FROM-16)

**DOI:** 10.1177/20552173251338762

**Published:** 2025-05-27

**Authors:** Rubina Shah, Sam Salek, Faraz M Ali, Kennedy Otwombe, Stuart J Nixon, Marie-Elaine Nixon, Gillian Ingram, John R Ingram, Andrew Y Finlay

**Affiliations:** Division of Infection and Immunity, School of Medicine, Cardiff University, Cardiff, UK; School of Life & Medical Sciences, 3769 University of Hertfordshire, Hatfield, UK; Institute of Medicines Development, Cardiff, UK; Division of Infection and Immunity, School of Medicine, Cardiff University, Cardiff, UK; Statistics and Data Management Centre, Perinatal HIV Research Unit, Chris Hani Baragwanath Academic Hospital, University of the Witwatersrand, Johannesburg, South Africa; School of Public Health, Faculty of Health Sciences, University of the Witwatersrand, Johannesburg, South Africa; Family members research partner, Cardiff UK; Department of Neurology, 7757 Swansea Bay University Health Board, Swansea, UK; Division of Infection and Immunity, School of Medicine, Cardiff University, Cardiff, UK

**Keywords:** Multiple sclerosis, family impact, quality of life, carer impact, family burden, Family-Reported Outcome Measure-16, FROM-16, spouse/partners, secondary impact

## Abstract

**Background:**

Multiple sclerosis (MS) may have a major impact on the physical, social and psychological wellbeing of people with multiple sclerosis (pwMS) and their family members/partners.

**Aim:**

To measure the impact of a person's MS on the quality of life of their family members/partner, and the associates of impact among family members, using a validated generic family-specific quality of life instrument, the Family Reported Outcome Measure (FROM-16).

**Methods:**

An online cross-sectional study was conducted to recruit family members/partners of pwMS through UK patient support groups.

**Results:**

A total of 219 family members/partners (mean age = 49.3 years, SD = 13.7; females = 55.3%) of pwMS (mean age = 50.1, SD = 12.5; females = 56.6%) completed the FROM-16. The FROM-16 mean total score was 16.9 (SD = 7.8), indicating ‘a very large effect’ on family members’ quality of life. The increasing age of pwMS, being a male person with MS, and being a female carer were significant predictors of family impact. 50.7% of family members had FROM-16 scores ≥17. Spouses/partners (170/219) of pwMS reported a significant impact on their sex life compared to other relationships (*p* < 0.001).

**Conclusion:**

MS substantially impacts the quality of life of family members/partners of pwMS, indicating a need to assess this impact routinely. The FROM-16 could be used to measure the MS family impact in routine practice to support family members appropriately and to include this impact in health economic appraisal and therapeutic clinical trials.

## Introduction

Multiple sclerosis (MS) is a chronic, progressive, immune-mediated neurodegenerative condition affecting the quality of life (QoL) of people with MS (pwMS) and their family members/partners.^1,2^ The prevalence of MS worldwide ranges from 5 to 300 per 100,000 people and may affect over 150,000 in the UK. MS often affects young adults, especially females, and causes various neurological problems, including with vision, balance, mobility, cognition, bladder and sexual functioning.^3^ Symptoms can progress, further disabling pwMS and increasing dependency on others, with significant limitation in walking by a mean age of 58.8 years.^4^ About 30% of pwMS need home assistance, of which 80% is provided by family members, primarily spouses/partners.^5^ This burden is important in health economic appraisals, with informal care costs estimated to account for 15% of the societal cost of MS.^6^

Caring for people with chronic conditions,^7^ including for pwMS,^1,8^ often leads to considerable carer burden. However, conceptualising and quantifying ‘burden’ is difficult, owing to various definitions and available measures; for example, researchers have measured MS carer burden using the Zarit burden scale,^8^ the CareQoL-MS^9^ and caregiver strain index (CSI),^10^ confusing understanding of the true MS burden. The consensus is that chronic diseases can have a negative impact on carer wellbeing and QoL.^11^ Most QoL instruments are based on a multidimensional conceptual approach, including physical, mental, social and functional aspects of health.^7^ Therefore, QoL instruments could provide a wider encompassing measure of carer impact in MS than just ‘burden’. As informal care in MS is mainly provided by family members,^2^ it would be appropriate to use family QoL instruments, covering all aspects of the impact of a person's chronic disease on family members. The use of standardised generic family QoL measures would allow a comparison of the QoL impact experienced by MS carers with other chronic illness groups. This study aims to measure the impact of a person's MS on the QoL of their family members/partner, and the associates of impact among family members, using a validated generic family-specific QoL instrument, the Family Reported Outcome Measure (FROM-16).^12^

## Materials and methods

### Study design and participants

This online cross-sectional study was conducted between April and November 2021 and involved family members and partners of pwMS recruited through the MS Trust, MS Society, Healthwise Wales (HWW) and Social Services Departments (SSDs) in Wales. Ethical approval was given by the Cardiff University School of Medicine Research Ethics Committee (SREC reference: 21/19).

Participants were recruited by convenience sampling. The study was open to UK family members/partners of pwMS aged ≥18 years and capable of operating electronic devices and providing written informed consent. Exclusion criteria included family members of deceased patients, family members <18 years, not capable of using electronic devices and family members not living in the UK. Family members/partners chose whether to participate after reading the participant information sheet embedded in the online questionnaire.

### Assessment instrument

#### FROM-16

MS family impact was assessed with FROM-16, which measures the impact of any disease on the QoL of adult family members or partners of patients of any age.^12^ FROM-16 comprises 16 items, each with three responses: ‘Not at All’ (scoring 0), ‘A Little’ (scoring 1) or ‘A Lot’ (scoring 2). The 16 items are divided into two categories (domains): Emotional (six items, maximum score = 12) and Personal and Social Life (10 items, maximum score = 20). The key themes include emotional impact (feeling of being worried, angry, sad, frustrated, difficulty caring and sharing thoughts) and personal and social impact (time for self, impact on travel, eating habits, family activities, holidays, sex life, work and study, family relationships, financial impact and sleep). The FROM-16 score range is 0–32. The higher the score, the greater the impact on the family member's QoL.

FROM-16 development was initially based on interviews with 133 family members of patients across 26 medical specialties. FROM-16 has high internal consistency (*n* = 120, Cronbach's α = 0.91) and high reproducibility (*n* = 51, ICC = 0.93), with a mean completion time of 2 minutes.^12^ Construct validity was confirmed through the correlation between FROM-16 and WHOQOL-BREF total scores (*n* = 119, *r* = −0.55, *p* < 0.001), and the correlation between FROM-16 and the patient's overall health score (*n* = 120, *r* = −0.51, *p* < 0.001)^12^ as well as the establishment of its longitudinal validity.^13^ Score interpretation is by validated score meaning bands^14^ and the Minimal Important Change value of FROM-16 is four points.^13^ FROM-16 has been mapped to EQ-5D-3L^15^ to allow the inclusion of family impact of disease in health economic appraisals.

### Procedure

The methodology and data presented in this study are part of a wider online study previously reported.^14^

The online study questionnaire had two sections: (1) pwMS completed basic information (gender, age, occupation, health condition and country of residence) about themselves and gave permission for their family member/partner to contribute to the study; (2) the family member/partner of pwMS completed basic demographic questions (gender, age, occupation and relationship to patient) and FROM-16.

There were two questionnaire formats: ‘patient and family member’ or ‘family member only’. The former was directed to patients registered with MS Society and MS Trust, while the latter was used with HWW and SSDs, for which patient information was completed by the family member/partner.

### Patient and public involvement

One MS patient and his spouse were study research partners, actively involved in the study design, participating in research team meetings and reviewing all study material.

### Data analysis

Descriptive analysis included calculating the mean, median, standard deviation and interquartile range of continuous variables and frequency and proportion for categorical variables. Nonparametric Mann-Whitney U-test and Kruskal-Wallis tests were used for group comparisons. Type I error probability was set at *p* < 0.05. FROM-16 descriptive banding was used to describe the severity of the impact of a person's MS on family members/partners. Multiple linear regression was used to investigate relationships between FROM-16 total score and patient age, family member age, family member gender (female, male, or other), family member's occupation (paid work, unemployed or retired) and relationship status (spouse, parent, adult children and others). Data analysis used IBM SPSS Statistics for Windows, version 27.

## Results

### Sociodemographic characteristics of study participants

A total of 219 family members/partners (mean age = 49.3 years, SD = 13.7, median = 50, range = 18–80; females = 55.3%) of pwMS (mean age = 50.1, SD = 12.5, median = 50, range = 19–87; females = 56.6%) completed FROM-16 (Table 1). Family members/partners were mostly from England (58.4%) and Wales (28.8%), with 22.4% retired, 55.3% in paid jobs and 7.3% in part-time jobs (Table 1). Family members were mostly spouses/partners of the pwMS (77.6%), followed by adult children (11%) (Table 1).

**Table 1.** Descriptive and sociodemographic characteristics.

Characteristics	Categories	Person with MS (*n* = 219) *N* (SD) or *N* (%)	Family member/partner (*n* = 219) *N* (SD) or *N* (%)
Age (years)	Mean (SD)	50.13 (12.5)	49.32 (13.7)
	Median (IQR)	50 (59–41)	50 (60–39)
	Range	19–87	18–80
Gender	Male	95 (43.4%)	96 (43.8%)
	Female	124 (56.6%)	121 (55.3%)
	Prefer not to say	0	1 (0.5%)
	Other	0	1 (0.5%)
Occupation	Full-time employment	73 (33.3%)	121 (55.3%)
	Part-time job	14 (6.4%)	16 (7.3%)
	Unemployed	37 (16.9%)	10 (4.6%)
	Education/training	1 (0.5%)	5 (2.3%)
	Homemaker	14 (6.4%)	17 (7.8%)
	Retired	74 (33.8%)	49 (22.4%)
	Rather not say	6 (2.7%)	1 (0.5%)
Country of residence	England	128 (58.4%)	
	Northern Ireland	4 (1.8%)	
	Scotland	24 (11%)	
	Wales	63 (28.8%)	
Relationship to patient	Spouse/partner		170 (77.6%)
	Parent		17 (7.8%)
	Adult child		24 (11.0%)
	Other (sibling, father/mother-in-law, son/daughter-in-law)		8 (3.7%)

MS: multiple sclerosis; SD: standard deviation; IQR: interquartile range.

The FROM-16 mean total score was 16.9/32 (SD = 7.8, median = 17, IQR = 12; emotional domain = 7.5/12, SD = 3.0; personal and social domain = 9.4/20, SD = 5.6). Of the individual FROM-16 items, ‘being worried’ had the highest mean score of 1.49 (SD = 0.5, max = 2), followed by ‘feeling frustrated’ (mean = 1.37, SD = 0.6, max = 2), ‘effect on family activities’ (mean = 1.37, SD = 0.7, max = 2), ‘feeling sad’ (mean = 1.35, SD = 0.6, max = 2). Impacts on ‘holiday’, ‘talking about thoughts’, ‘sex life’, ‘sleep’, ‘difficulty caring,’ and ‘effect on family expenses’ had the next highest mean scores (Table 2).

**Table 2.** Mean total FROM-16 scores and individual item scores of family members/partners of pwMS (*n* = 219).

FROM-16	Description	Mean (SD)	Median (IQR)	Range
Total FROM-16 mean score	Overall	16.88 (7.8)	17 (23-11)	0–32
Domain score	Emotional domain	7.45 (3.0)	8 (10-5)	0–12
	Personal and social domain	9.43 (5.6)	9 (14-5)	0–20
FROM-16 individual items score	Worried	1.49 (0.5)	2 (2-1)	0–2
	Angry	0.96 (0.7)	1 (1-0)	0–2
	Sad	1.35 (0.6)	1 (2-1)	0–2
	Frustrated	1.37 (0.6)	1 (2-1)	0–2
	Talking about thoughts	1.21 (0.8)	1 (2-1)	0–2
	Difficulty caring	1.07 (0.7)	1 (2-1)	0–2
	Time for self	1.03 (0.8)	1 (2-1)	0–2
	Everyday travel	0.54 (0.8)	0 (1-0)	0–2
	Eating habits	0.60 (0.7)	0 (1-0)	0–2
	Family activities	1.37 (0.7)	2 (2-1)	0–2
	Holiday	1.24 (0.8)	1 (2-1)	0–2
	Sex life	1.11 (0.8)	1 (2-1)	0–2
	Work or study	0.69 (0.8)	0 (1-0)	0–2
	Family relationships	0.75 (0.8)	1 (1-0)	0–2
	Family expenses	1.00 (0.8)	1 (2-0)	0–2
	Sleep	1.09 (0.8)	1 (2-0)	0–2

pwMS: people with multiple sclerosis; FROM-16: Family Reported Outcome Measure-16; SD: standard deviation; IQR: interquartile range.

**Figure 1.**
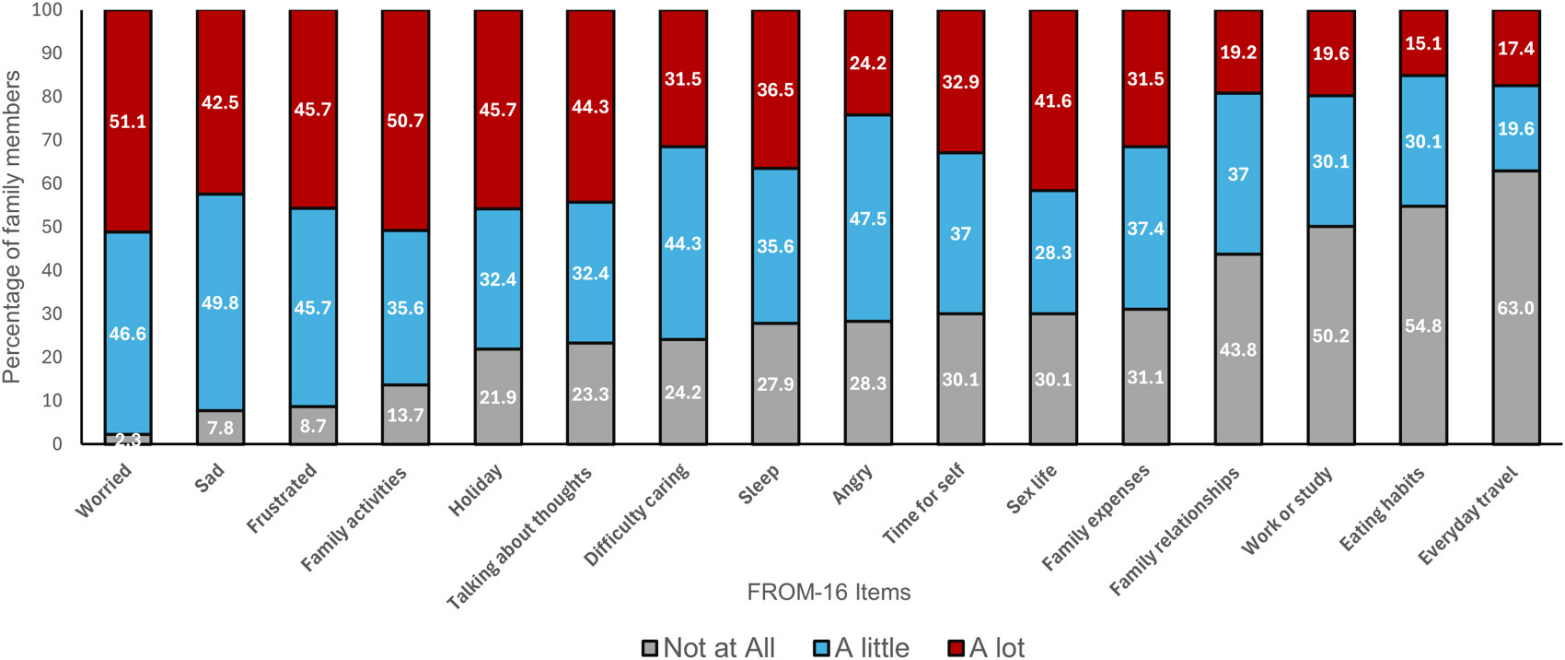
Impact of relative's MS on family members/partners across the 16 items of FROM-16.

MS: multiple sclerosis; FROM-16: Family Reported Outcome Measure-16.

The most frequently reported impacts on the QoL of family members/partners included feeling worried (97.7%), feeling sad (92.3%), feeling frustrated (91.4%), impacts on family activities (86.3%), holidays (78.1%), talking about thoughts (76.7%), difficulty caring (75.8%) and impact on sleep (72.1%) (Figure 1).

Cronbach's α for this study data was carried out, which yielded a value of 0.91, confirming the internal consistency reliability of the FROM-16.

### Contextualising study participants’ QoL using FROM-16 severity score bands

The study explored the degree of severity of impact experienced by family members/partners^14^: 50.6% had FROM-16 scores ≥17, indicating ‘a very large’ or ‘extremely large’ impact. Only one family member experienced ‘no impact’ (Table 3).

**Table 3.** FROM-16 score banding^14^ describing the impact of MS on the quality of life of family members/partners (*n* = 219).

FROM-16 score banding	Number of family members	Percentage of family members
No effect (0–1)	1	0.5
A small effect (2–8)	37	16.9
A moderate effect (9–16)	70	32.0
A very large effect (17–25)	73	33.3
An extremely large effect (26–32)	38	17.3
Total	219	100

MS: multiple sclerosis; FROM-16: Family Reported Outcome Measure-16.

### Comparison of family members/partner QoL by gender

Female family members of pwMS experienced more impact from their relative's MS than males (*p* < 0.001). This difference occurred across all individual FROM-16 items except for the ‘effect on everyday travel’ (Supplemental Table S1).

### Impact of a person's MS across family relationships

Adult children (*n* = 24) had a higher mean FROM-16 score (18.8, SD = 7.6) compared to other relations (spouse/partners (*n* = 177): mean = 16.7, SD = 7.8; parents (*n* = 17): mean = 17.5, SD = 8.8; others (*n* = 8): mean = 14, SD = 7.2), but these differences were not statistically significant (Supplemental Table S2).

There were significant differences in impact between various family relations across the 16 individual items of FROM-16 (Supplemental Figure S1). Spouses/partners of PwMS experienced a significant impact on their sex life compared to other carers (*p* < 0.001). Parents experienced more frustration and anger than spouses/partners (*p* < 0.05). Compared to spouses/partners, adult children felt significantly more sad, angry and worried, experienced a significant impact on family relationships (*p* < 0.05) and significant difficulties in caring (*p* < 0.01). Adult children also experienced significantly more impact on their ‘work/study’ than all other relations (*p* < 0.05) (Supplemental Table S3). There was no significant difference in total FROM-16 scores between the different relationships with respect to country of residence and family members’ occupation (Supplemental Table S4a and b).

### Multiple linear regression – Factors predicting outcomes

Multiple regression analysis showed a significant relationship between the FROM-16 total score and pwMS age (*p* < 0.001), pwMS gender (*p* < 0.001) and family member gender (*p* = 0.04). The family impact of MS (FROM-16 total score) increased with increasing age of pwMS: on average, a one-year age increase resulted in a 0.164 increase in the FROM-16 score (*p* = 0.001). The FROM-16 score varied according to family members and pwMS gender. Compared to females, male pwMS predicted higher FROM-16 scores (*p* < 0.001). Also compared to male family members/partners, females had higher FROM-16 scores than male family members. Females had a 2.4-point higher mean FROM-16 score than males. Model fit statistics were done using collinearity diagnostics, and all were within the acceptable threshold (tolerance > 0.5 and variance inflation factor (VIF) < 2). The multiple correlation coefficient squared (*R*^2^) represents the proportion of the variance in the dependent variable accounted by the independent variables. The *R*^2^ was 0.28, suggesting 28% of the variance was explained by the model containing the FROM-16 total score, pwMS and family members’ age and gender, family member occupation and family member relationship to the pwMS (Table 4).

**Table 4.** Factors associated with the family impact of MS (univariate and multivariate analyses).

Variables	Univariate				Multivariate			
	*B*	Lower 95%	Upper 95%	*p*-value	*B*	Lower 95%	Upper 95%	*p*-value
FM age	0.31	−0.045	0.107	0.425	–	–	–	
pwMS age	0.169	0.089	0.250	<0.001	0.164	0.085	0.243	<0.001
FM gender								
Female vs. male	6.009	4.074	7.944	<0.001	2.402	0.066	4.738	0.044
pwMS gender								
Female vs. male	−6.754	−8.652	−4.856	<0.001	−4.679	−7.007	−2.350	<0.001
FM occupation								
Unemployed vs. paid work	3.277	0.280	6.274	0.032	2.504	−0.120	5.129	0.061
Retired vs. paid work	−0.177	−2.717	2.364	0.891	−2.046	−4.442	0.349	0.094
FM relationship								
Adult child vs. spouse/partner	2.151	−1.205	5.507	0.208				
Parent vs. spouse/partner	0.788	−3.126	4.703	0.692				
Others vs. spouse/partner	−2.682	−8.250	2.885	0.343				

Dependent variable: FROM-16 summary score; *R*^2^ = 28%; tolerance > 0.5 and VIF < 2; significance at 5%. FM: family member; pwMS: people with multiple sclerosis; Not included: two family members did not specify gender, and one family member did not specify occupation; MS: multiple sclerosis; FROM-16: Family Reported Outcome Measure; VIF: variance inflation factor.

## Discussion

MS significantly impacts families, influencing their wellbeing and QoL, often affecting each family member and family dynamic.^2^ Our study has confirmed the substantial impact of a person's MS on their family members/partners, using the validated generic family QoL instrument, the FROM-16.

More than 50% of family members/partners had FROM-16 scores ≥17, indicating ‘a very large’ or ‘extremely large’ impact of a relative's MS on the QoL of family members/partners.^14^ This level of QoL impairment should trigger engagement in family support services. Only 0.5% of family members/partners reported no effect of their relative's MS on their QoL, confirming that almost everyone is impacted.

Family members/partners of pwMS experienced a huge emotional impact, with 97.7% reporting feeling worried, 92.3% feeling sad and 91.4% feeling frustrated. This could be attributed to the unpredictable nature of MS, with family members, particularly spouses/partners, having to face considerable lifestyle and role adjustments that can cause them emotional distress and reduce their QoL.^8^ Janssens et al.^16^ reported that around 50% of MS patients and their partners experienced a substantial emotional burden soon after the diagnosis, with higher levels of anxiety and distress than the general population. Giordano et al.^17^ reported MS carers experienced lower QoL compared to non-carers, with 68% of MS carers recording pathological anxiety and 44% pathological depression. In an Italian study,^8^ 84.0% of family members reported feeling worried about the patient's future, with 82.5% feeling worried about fulfilling various responsibilities. In another study,^18^ MS caregivers described feeling frustrated and impatient about the effects of cognitive impairment on the relative's functioning when using the computer, shopping, or managing bills.

In our study, three of four family members/partners reported difficulty caring for their relatives and finding someone to talk to about their thoughts. This is not surprising given the unpredictable nature of MS and its debilitating impact, with family members/partners having less time to maintain social connections, leading to alienation.^19^

Caring for a relative with a chronic condition can impact family activities and social life.^7^ In our study, 86.3% of family members/partners of pwMS reported that their relatives’ MS impacted their family activities and 78.1% reported an impact on holidays, presumably related to the physical and emotional demands and the time-intensive nature of caring. Halstead et al.^18^ reported that many couples rescheduled or cancelled social events due to fatigue exacerbating cognitive symptoms.

Caring can impact sleep, leading to immune suppression^20^ and affecting physical wellbeing. In our study, 72.1% of family members/partners reported their relative's MS impacted their sleep. This is higher than a Greek study,^21^ where 54.3% of MS caregivers reported sleep disorders, but is comparable to that reported by family members/partners of people with dementia (81.4%).^22^ Poor sleep may be caused by caregiving stress and worry or by disruptions at night from the patient's MS symptoms. Poor sleep quality in MS family caregivers has been associated with increased levels of anxiety (*r* = 0.392; *p* = 0.02) and depression (*r* = 0.424; *p* = 0.01).^21^

Caring for a relative with MS also affects the financial situation of family members/partners. Consistent with other studies,^23^ 68.9% of family members in our study reported that their relative's MS impacted their family expenses. The pwMS and/or their family members/partners may have to leave paid employment or work fewer hours, reducing family income.^24,25^ In our study, 33.3% of pwMS and 55.3% of family members/partners were in full-time paid jobs, while 6.4% of pwMS and 7.3% of family members/partners were in part-time paid jobs. However, of those working-age (18–65 years), 37.4% of pwMS and 62.4% of family members/partners were full-time, while 7.2% of pwMS and 8.2% of family members/partners were in part-time paid jobs.

Although the difference in overall FROM-16 scores was not significantly different between family relations, there were some significant differences between family members with respect to individual FROM-16 items. Spouses/partners of pwMS reported a significant impact on their sex life. As most family members (77.6%) in this study were spouses/partners, this is one of the key findings of this study, with female spouses/partners reporting greater impact, which is consistent with findings of an Iranian study.^26^ In our study, adult children experienced significantly more impact on their emotional health and family relationships (*p* < 0.05) than spouses/partners. This may be because adult children find it stressful to juggle competing responsibilities and balance the highly demanding caring needs of their MS parents with attending to other family members. Adult children were impacted significantly more across ‘work/study’ than spouses/partners, parents and other relationships (*p* < 0.05).

Female family members of pwMS experienced more negative QoL impact than males across all FROM-16 items, except for the ‘effect on everyday travel’, which may have been biased as the study was conducted during the COVID-19 pandemic. Being a female carer was a significant predictor of the family impact of MS, perhaps because the complex and demanding role of women in the family may lead to increased burden and stress.^27^ Another potential reason could be that men differ from women in their concepts of awareness of problems and in their willingness to report problems and communicate with friends and the care team.^28^

### Comparisons with FROM-16 studies involving other chronic conditions

Our study findings demonstrate that the family impact of MS (mean FROM-16 score = 16.9) is comparable to that of dementia (mean FROM-16 score = 17.5)^22^ and myalgic encephalomyelitis/chronic fatigue syndrome (mean FROM-16 score = 17.9)^29^ but higher than that of family impact of COVID-19 (mean FROM-16 score = 15.0),^30^ diabetes (mean FROM-16 score = 10.5)^31^ and haematological conditions (mean FROM-16 score = 14.0),^32^ all of which were conducted during the COVID-19 pandemic. The family impact of MS in this study is much higher than other FROM-16 studies conducted before the pandemic, reporting the family impact of chronic conditions across 26 medical specialties (mean FROM-16 score = 12.4)^33^ and family impact of malignancies (mean FROM-16 score = 11.8).^34^

### Study limitations

As the study was conducted during the COVID-19 pandemic, the results could have been influenced by the pandemic impact on pwMS and their family members/partners. However, our findings are consistent with other MS studies conducted pre-pandemic. The study was conducted online, with participants recruited through patient support groups. This may have resulted in recruiting the most motivated participants and capable of using electronic devices, thereby introducing selection bias. However, this was the only way to reach out to participants during the pandemic. In our sample, though there were more female than male pwMS, the proportion of females was less than population norms. Furthermore, this study did not collect data on ethnicity or race, and it is not possible to comment on the sample diversity.

When completing the survey, the pwMS answered first and then the family member/partner. The family member was, therefore, able to view the responses of the pwMS (which only included patient demographic information), but the pwMS would not have been able to view the responses of the family member unless the family member allowed this. Although this appears to raise potential ethical concerns over the potential sharing of information between the pwMS and their family member, the survey format was approved by the ethics committee.

MS is a highly variable disease with some pwMS experiencing little persistent symptoms, and some having a severe disability. Due to the study's self-reported enrolment, data on disease course, duration and disability status/type of MS was not available. However, the extent of disability is likely to have a large impact on the family members/carer's role. Associations found between increasing age of pwMS and family impact may be partly explained by this. For example, those with progressive MS are likely to be older with higher care needs. It would be useful to extend this study to a larger group of patients with linked clinical data, to understand the impact on carers at all disease stages. This study may have overestimated the effect on the QoL of carers if pwMS with higher levels of disability were more inclined to participate. Despite these limitations, this study provides important data on the impact of MS on UK family members and partners using a validated generic family-specific measure, the FROM-16.

### Implication for practice

Consistent with other findings,^2,8,21^ this study has demonstrated that family members/partners of pwMS experience a substantial negative impact on their QoL, which is often ignored and excluded from healthcare policy and economic appraisal. Poor health of MS family caregivers and the burden of MS caregiving increases the probability of nursing home placement,^5^ with a consequential rise in healthcare costs. Therefore, it is critical to measure the family impact of MS in routine practice using family-specific instruments, thus ensuring the wellbeing of family caregivers and continued support for pwMS. The FROM-16, with its well-established measurement properties, could be used in routine practice to identify and support impacted family members/partners of pwMs. Routinely collected FROM-16 data could be used to incorporate the family impact of MS in health economic evaluation.^15^ Furthermore, FROM-16, with established longitudinal validity, could be used to measure the family impact of disease-modifying treatment in real-world practice and clinical trials.

Consistent with other studies, one of the key findings of our work was that the spouses/partners of pwMS reported a significant impact on their sex life, which can have a damaging effect on couple relationships^35^ and consequently on patient care. Therefore, this study should inform health policy and clinical interventions, such as relationship counselling.

## Conclusion

This study has shown the substantial impact of a person's MS on their family members and partners. There is a need to measure this impact in routine practice to identify family members needing support, thereby ensuring holistic clinical practice and strong family support for MS patients.

## Supplemental Material

sj-pdf-1-mso-10.1177_20552173251338762 - Supplemental material for Multiple sclerosis greatly impacts family members/partners: Evidence using the Family Reported Outcome Measure (FROM-16) Supplemental material, sj-pdf-1-mso-10.1177_20552173251338762 for Multiple sclerosis greatly impacts family members/partners: Evidence using the Family Reported Outcome Measure (FROM-16) by Rubina Shah, Sam Salek, Faraz M Ali, Kennedy Otwombe, Stuart J Nixon, Marie-Elaine Nixon, Gillian Ingram, John R Ingram and Andrew Y Finlay in Multiple Sclerosis Journal – Experimental, Translational and Clinical

## References

[1] Opara J Brola W . Quality of life and burden in caregivers of multiple sclerosis patients. Physiother Health Activity 2018; 25: 16–19.

[2] Uccelli MM . The impact of multiple sclerosis on family members: a review of the literature. Neurodegener Dis Manag 2014; 4: 177–185. 24832035 10.2217/nmt.14.6

[3] Brownlee WJ Hardy TA Fazekas F , et al. Diagnosis of multiple sclerosis: progress and challenges. Lancet 2017; 389: 1336–1346. 27889190 10.1016/S0140-6736(16)30959-X

[4] Harding KE Ingram G Tallantyre EC , et al. Contemporary study of multiple sclerosis disability in South East Wales. J Neurol Neurosurg Psychiatry 2023; 94: 72. 10.1136/jnnp-2022-330013 36328420

[5] Buchanan RJ Radin D Huang C , et al. Caregiver perceptions associated with risk of nursing home admission for people with multiple sclerosis. Disabil Health J 2010; 3: 117–124. 21122777 10.1016/j.dhjo.2009.08.003

[6] Adal TG van der Mei I Taylor BV , et al. Investigation of the health economic analysis of informal care for people living with a chronic neurological disease: a systematic review and meta-analysis of the global evidence for multiple sclerosis. Soc Sci Med 2024; 363: 117405. 39541831 10.1016/j.socscimed.2024.117405

[7] Shah R Ali FM Finlay AY , et al. Family reported outcomes, an unmet need in the management of a patient's disease: appraisal of the literature. Health Qual Life Outcomes 2021; 19: 194. 2021/08/07. 34353345 10.1186/s12955-021-01819-4 PMC8339395

[8] Ponzio M Tacchino A Verri A , et al. Profile and burden of the family caregiver: the caring experience in multiple sclerosis. An observational study. BMC Psychol 2024; 12: 73. 38528601 10.1186/s40359-024-01678-w PMC10964650

[9] Benito-León J Rivera-Navarro J Guerrero AL , et al. The CAREQOL-MS was a useful instrument to measure caregiver quality of life in multiple sclerosis. J Clin Epidemiol 2011; 64: 675–686. 2010/11/13. 21071173 10.1016/j.jclinepi.2010.08.003

[10] Robinson BC . Validation of a caregiver strain index. J Gerontol 1983; 38: 344–348. 1983/05/01. 6841931 10.1093/geronj/38.3.344

[11] Maguire R Maguire P . Caregiver burden in multiple sclerosis: recent trends and future directions. Curr Neurol Neurosci Rep 2020; 20: 18. 2020/05/24. 32444986 10.1007/s11910-020-01043-5 PMC7242779

[12] Golics CJ Basra MK Finlay AY , et al. The development and validation of the family reported outcome measure (FROM-16)© to assess the impact of disease on the partner or family member. Qual Life Res 2014; 23: 317–326. 2013/06/26. 23797564 10.1007/s11136-013-0457-y

[13] Shah R Finlay AY Salek SM , et al. Responsiveness and minimal important change of the Family Reported Outcome Measure (FROM-16). J Patient Rep Outcomes 2023; 8(1): 38. 10.1186/s41687-024-00703-1 PMC10965873 38530614

[14] Shah R Finlay AY Salek SM , et al. Meaning of family reported outcome measure (FROM-16) severity score bands: a cross-sectional online study in the UK. BMJ Open 2023; 13: e066168. 2023/03/24. 10.1136/bmjopen-2022-066168 PMC10040025 36958787

[15] Shah R Salek S Finlay AY , et al. Mapping of family reported outcome measure (FROM-16) scores to EQ-5D: algorithm to calculate utility values. Qual Life Res 2024: 1–13. DOI: 10.1007/s11136-023-03590-z PMC10973087 38402530

[16] Janssens AC van Doorn PA de Boer JB , et al. Impact of recently diagnosed multiple sclerosis on quality of life, anxiety, depression and distress of patients and partners. Acta Neurol Scand 2003; 108: 389–395. 2003/11/18. 14616290 10.1034/j.1600-0404.2003.00166.x

[17] Giordano A Cimino V Campanella A , et al. Low quality of life and psychological wellbeing contrast with moderate perceived burden in carers of people with severe multiple sclerosis. J Neurol Sci 2016; 366: 139–145. 27288793 10.1016/j.jns.2016.05.016

[18] Halstead EJ Stanley J Fiore D , et al. Impact of cognitive impairment on adults with multiple sclerosis and their family caregivers. Int J MS Care 2021; 23: 93–100. 2021/06/29. 34177380 10.7224/1537-2073.2019-091 PMC8218589

[19] Carberry S MacConaill S Fortune DG . Couples’ experiences of coping with multiple sclerosis: a qualitative systematic review and metasynthesis. Disabil Rehabil 2024: 1–13. DOI: 10.1080/09638288.2024.2361804 38859675

[20] Garbarino S Lanteri P Bragazzi NL , et al. Role of sleep deprivation in immune-related disease risk and outcomes. Commun Biol 2021; 4: 1304. 2021/11/20. 34795404 10.1038/s42003-021-02825-4 PMC8602722

[21] Argyriou AA Karanasios P Assimakopoulos K , et al. Assessing the quality of sleep in Greek primary caregivers of patients with secondary progressive multiple sclerosis: a cross-sectional study. J Pain Symptom Manage 2011; 42: 541–547. 2011/03/30. 21444179 10.1016/j.jpainsymman.2011.01.005

[22] Shah R Salek MS Ali FM , et al. Dementia and its profound impact on family members and partners: a large UK cross-sectional study. Alzheimer Dis Assoc Disord 2024; 38: 338–343. 39506214 10.1097/WAD.0000000000000647 PMC11584184

[23] Karampampa K Gustavsson A Miltenburger C , et al. Treatment experience, burden and unmet needs (TRIBUNE) in MS study: results from five European countries. Mult Scler J 2012; 18: 7–15. 10.1177/1352458512441566 22623122

[24] Hendin B Brook RA Beren IA , et al. The clinical and economic impact of employees who are care partners of patients with multiple sclerosis by disease severity. J Health Econ Outcomes Res 2023; 10: 91–101. 2023/04/19. 10.36469/001c.57593 PMC10105615 37069893

[25] Hategeka C Traboulsee A Mcmullen K , et al. Association of unemployment and informal care with stigma in multiple sclerosis: evidence from the survey on living with neurological conditions in Canada. Int J MS Care 2019; 21: 214–225. 31680783 10.7224/1537-2073.2017-108 PMC6819020

[26] Sedighi B Abedini Parizi M Haghdoost AA , et al. How does multiple sclerosis affect sexual satisfaction in patients’ spouses? Front Psychol 2023; 14: 1110884. 2023/04/21. 37082576 10.3389/fpsyg.2023.1110884 PMC10111138

[27] Penning MJ Wu Z . Caregiver stress and mental health: impact of caregiving relationship and gender. Gerontologist 2016; 56: 1102–1113. 2015/06/03. 26035875 10.1093/geront/gnv038

[28] Robinson CA Bottorff JL Pesut B , et al. The male face of caregiving: a scoping review of men caring for a person with dementia. Am J Mens Health 2014; 8: 409–426. 24414033 10.1177/1557988313519671

[29] Vyas J Muirhead N Singh R , et al. Impact of myalgic encephalitis/chronic fatigue syndrome (ME/CFS) on the quality of life of people with ME/CFS and their partners and family members: an online cross-sectional study. BMJ Open 2022; 11: e058128. 10.1136/bmjopen-2021-058128 PMC9062824 35501074

[30] Shah R Ali FM Nixon SJ , et al. Measuring the impact of COVID-19 on the quality of life of the survivors, partners and family members: a cross-sectional international online survey. BMJ Open 2021; 11: e047680. 2021/05/27. 10.1136/bmjopen-2020-047680 PMC8154981 34035105

[31] Shah R Finlay AY Ali FM , et al. Comparison of the impact of Type 1 and Type 2 diabetes on quality of life of families of patients: a UK cross-sectional study. Diabetes Obes Metab 2025; 27: 652–662. 39582116 10.1111/dom.16058 PMC11701181

[32] Shah R Finlay AY Ali FM , et al. Measurement of the major ignored burden of multiple myeloma, pernicious anaemia and of other haematological conditions on partners and family members: a cross-sectional study. Eur J Haematol 2024; 113: 117–126. 38577720 10.1111/ejh.14206

[33] Golics CJ Basra MK Salek MS , et al. The impact of patients’ chronic disease on family quality of life: an experience from 26 specialties. Int J Gen Med 2013; 6: 787–798. 2013/10/05. 24092994 10.2147/IJGM.S45156 PMC3787893

[34] Chantarasap P Johns NP Pairojkul S , et al. Validation of the Thai version of the family reported outcome measure (FROM-16)© to assess the impact of disease on the partner or family members of patients with cancer. Health Qual Life Outcomes 2019; 17: 32. 30736795 10.1186/s12955-019-1091-3 PMC6368697

[35] Landfeldt E Castelo-Branco A Svedbom A , et al. The long-term impact of multiple sclerosis on the risk of divorce. Mult Scler Relat Disord 2018; 24: 145–150. 30007180 10.1016/j.msard.2018.07.002

