# Supplementary material for: Multiple sclerosis greatly impacts family members/partners: Evidence using the Family Reported Outcome Measure (FROM-16)
Source: Mult Scler J Exp Transl Clin. 2025 May 27;11(2):20552173251338762. doi: 10.1177/20552173251338762 (PMC12120303; doi:10.1177/20552173251338762)
Supplement: sj-pdf-1-mso-10.1177_20552173251338762 - Supplemental material for Multiple sclerosis greatly impacts family members/partners: Evidence using the Family Reported Outcome Measure (FROM-16) [file sj-pdf-1-mso-10.1177_20552173251338762.pdf]

Supplementary material

Multiple Sclerosis greatly impacts family members/partners: evidence using the Family Reported Outcome Measure (FROM-16)

INDEX: Tables/figures

Table S1. Comparisons[†] of family impact of person's MS based on family members' gender (n=219)

Table S2. Family Impact of MS across the relationships (n=219)

Figure S1. Comparison of the family impact of MS on relationships across FROM-16 items (n=219)

Table S3. Comparison[T] of impact across FROM-16 items between family relationships

Table S4a. Comparison[T] of impact across family relationships with respect to country of residence

Table S4b. Comparison[T] of impact across family relationships with respect to pwMS's occupation

Table S1 Comparisons† of family impact of person's MS based on family members' gender (n=219)

FROM-16 Items	Gender¥ Mean score (SD)		p-value
	Male (n=96)	Female (n=121)	
**Overall**	13.23 (7.0)	19.57 (7.2)	< 0.001
**Emotional domain**	6.05 (2.9)	8.48 (2.6)	< 0.001
**Personal and Social domain**	7.18 (4.5)	11.10 (5.3)	< 0.001
**Worried**	1.34 (0.6)	1.60 (0.5)	0.001
**Angry**	0.67 (0.6)	1.17 (0.7)	< 0.001
**Sad**	1.09 (0.6)	1.54 (0.5)	< 0.001
**Frustrated**	1.17 (0.8)	1.52 (0.6)	< 0.001
**Talking about thoughts**	0.93 (0.8)	1.42 (0.7)	< 0.001
**Difficulty caring**	0.85 (0.7)	1.23 (0.7)	< 0.001
**Time for self**	0.75 (0.8)	1.24 (0.8)	< 0.001
**Everyday travel**	0.46 (0.7)	0.60 (0.8)	0.223
**Eating habits**	0.42 (0.6)	0.74 (0.8)	0.001
**Family activities**	1.13 (0.7)	1.55 (0.7)	< 0.001
**Holiday**	0.98 (0.8)	1.43 (0.7)	< 0.001
**Sex life**	0.96 (0.7)	1.22 (0.9)	0.012
**Work or study**	0.42 (0.6)	089 (0.8)	< 0.001
**Family relationships**	0.43 (0.6)	0.99 (0.7)	< 0.001
**Family expenses**	0.84 (0.8)	1.12 (0.8)	0.012
**Sleep**	0.80 (0.7)	1.30 (0.8)	< 0.001

† Mann Whitney U test; *p ≤ 0.05, **p ≤ 0.01, 2-tailed. (p values were calculated using mean rank scores, but mean scores are presented here for ease of understanding);¥One family member identified as 'other' had a FROM-16 score =27, and one other family member who did not want to mention gender had a FROM-16 score=32.

Table S2 Family Impact of MS across the relationships (n=219)

FROM-16	Spouses/partners (n=170)	Parents (n=17)	Adult children (n=24)	Other (n=8)	p-value
Mean(SD)	16.68 (7.8)	17.47 (8.8)	18.83 (7.6)	14 (7.2)	0.434
Median	16	19	20	13	
Range	1-32	4-31	4-31	4-25	
FM age Mean (SD)	50.6 (12.2)	60.4 (11.8)	33.3 (12.1)	46.5 (13.9)	
Patient age Mean (SD)	50.2 (11.2)	40.1 (14.9)	58.0 (11.8)	45.4 (14.3)	

Kruskal Walis test ; significance at 0.05.

Figure S1 Comparison of the family impact of MS on relationships across FROM-16 items (n=219)

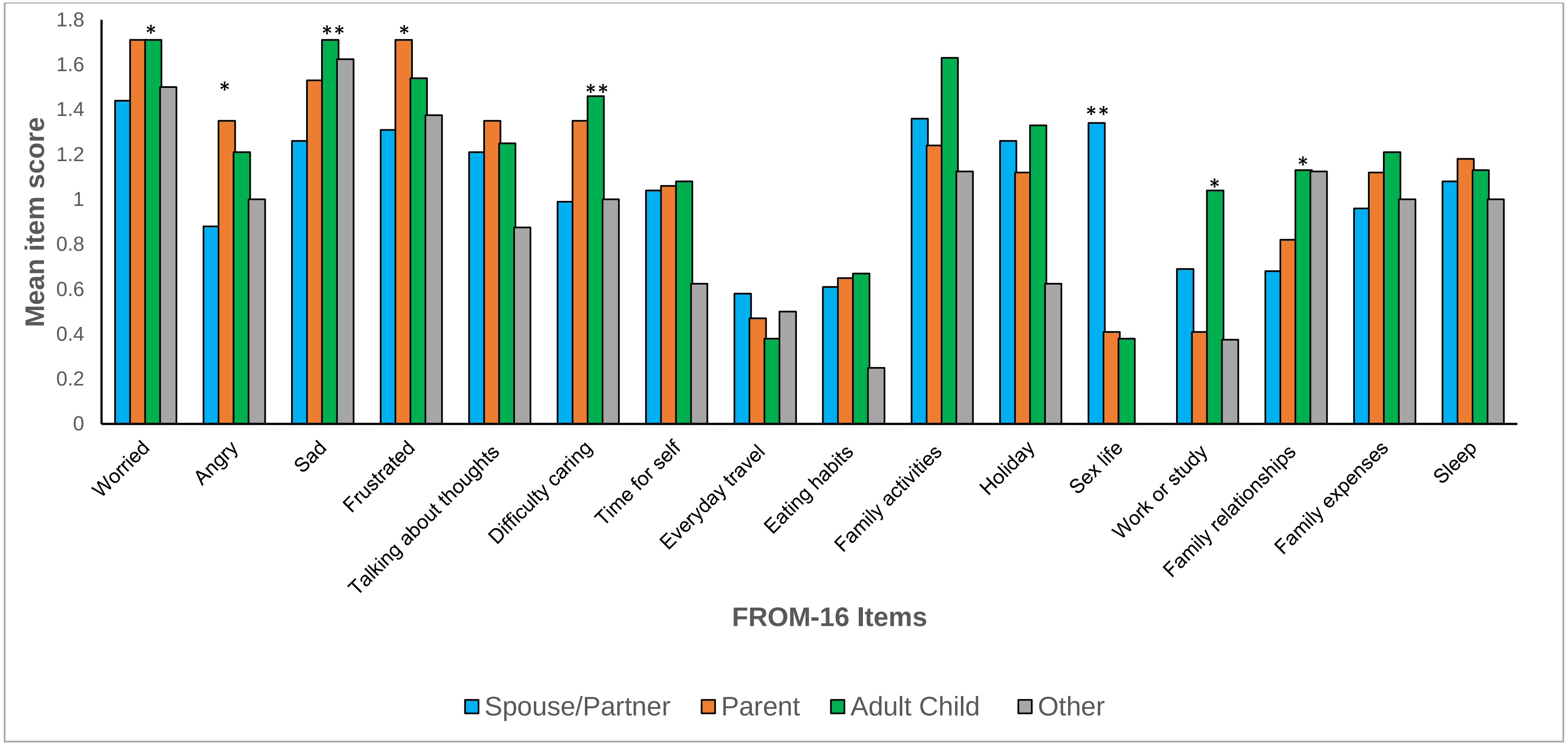

*p ≤ 0.05, **p ≤ 0.01, 2-tailed.

Table S3. Comparison[T] of impact across FROM-16 items between family relationships

FM- relationship comparison		Sex life	Sad	Difficulty caring	Angry	Family relationships	Work or study	Frustrated	Worried
		Test statistics	Test statistics	Test statistics	Test statistics	Test statistics	Test statistics	Test statistics	Test statistics
Pairwise Comparisons of FM-relationship	Spouse /Partner-Adult children	66.00**	-41.01**	-37.08**	-26.48*	-32.73*	-24.65	-18.70	-27.64*
	Spouse/Partner-Parent	64.26**	-22.94	-28.61	-37.87*	-12.54	22.16	-37.51*	-27.38
	Spouse/Partner-Other	92.09**	-32.59	-2.37	-9.57	21.24	25.64	-2.04	-5.35
	Parent-Adult children	-1.74	18.07	8.47	-11.39	20.20	46.81*	-18.80	0.26
	Other-Adult children	26.08	8.42	34.71	16.92	-4.50	50.29*	16.67	22.29
	Other-Parent	27.82	-9.65	26.24	28.30	-24.70	3.48	35.47	22.03

[T]Kruskal-Wallis-I way ANOVA Test. Each row tests the null hypothesis that the Sample 1 and Sample 2 distributions are the same. Asymptotic significances (2-sided tests) are displayed. The significance level is assumed when the p-value <0.05. No significant differences were found between FM-relationship (Spouse/partner, adult children, parent, and other) with respect to the following FROM-16 items: Holiday, Family activities, Everyday travel, Family expenses, Time for self, Talking about thoughts, Eating habits and Sleep. Therefore, no further analysis was done for these items. *p ≤ 0.05, **p ≤ 0.01, 2-tailed.

Table S4a. Comparison[T] of impact across family relationships with respect to country of residence

Residence	FM Relationship	N	Mean Rank	p-value
**England**	Spouse / Partner	104	64.3	0.931
	Adult children	12	68.88	
	Parent	8	65.31	
	Other	4	54.88	
	Total	128		
**Northern Ireland**	Spouse / Partner	1	1	0.407
	Adult children	1	3	
	Parent	2	3	
	Total	4		
**Scotland**	Spouse / Partner	20	13.3	0.539
	Adult children	2	10.75	
	Parent	1	8.5	
	Other	1	4	
	Total	24		
**Wales**	Spouse / Partner	45	30.11	0.420
	Adult children	9	41.22	
	Parent	6	33.25	
	Other	3	30.17	
	Total	63		

[T]Kruskal-Wallis Test. Asymptotic significances (2-sided tests), the significance level is p-value <0.05.

Table S4b. Comparison[T] of impact across family relationships with respect to pwMS's occupation

**pwMS Occupation**	FM Relationship	N	Mean Rank	p-value
**Unemployed**	Spouse / Partner	32	26.23	0.863
	Adult children	10	28.45	
	Parent	7	27.64	
	Other	3	20.17	
	Total	52		
**Full-time employment**	Spouse / Partner	58	37.34	0.43
	Adult children	4	50.38	
	Parent	8	31.44	
	Other	3	27.5	
	Total	73		
**Part-time job**	Spouse / Partner	14	7.5	
	Total	14[a]		
**Retired**	Spouse / Partner	61	37.07	0.288
	Adult children	10	35	
	Parent	2	66	
	Other	1	31.5	
	Total	74		
**Not specified**	Spouse / Partner	5	3.7	0.546
	Other	1	2.5	
	Total	6		

[T]Kruskal-Wallis Test. [a] There is only one non-empty group, so the Kruskal-Wallis Test could not be performed. Asymptotic significances (2-sided tests), the significance level is p-value <0.05. One FM did not specify occupation and is not included in the above analysis.
